# Controlling gene expression with light: a multidisciplinary endeavour

**DOI:** 10.1042/BST20200014

**Published:** 2020-07-13

**Authors:** Denis Hartmann, Jefferson M. Smith, Giacomo Mazzotti, Razia Chowdhry, Michael J. Booth

**Affiliations:** Chemistry Research Laboratory, University of Oxford, Oxford OX1 3TA, U.K.

**Keywords:** biochemical techniques and resources, biotechnology, gene expression and regulation

## Abstract

The expression of a gene to a protein is one of the most vital biological processes. The use of light to control biology offers unparalleled spatiotemporal resolution from an external, orthogonal signal. A variety of methods have been developed that use light to control the steps of transcription and translation of specific genes into proteins, for cell-free to *in vivo* biotechnology applications. These methods employ techniques ranging from the modification of small molecules, nucleic acids and proteins with photocages, to the engineering of proteins involved in gene expression using naturally light-sensitive proteins. Although the majority of currently available technologies employ ultraviolet light, there has been a recent increase in the use of functionalities that work at longer wavelengths of light, to minimise cellular damage and increase tissue penetration. Here, we discuss the different chemical and biological methods employed to control gene expression, while also highlighting the central themes and the most exciting applications within this diverse field.

## Introduction

**Figure 1.**
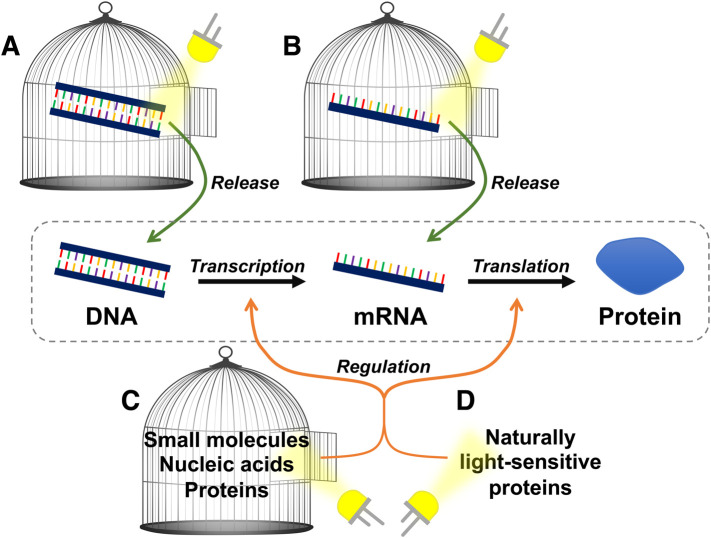
Methods of controlling transcription and translation with light discussed in this review. Uncaging the (**A**) DNA or (**B**) mRNA template of a gene of interest with light allows for activation of transcription/translation. Whereas regulation of gene expression with light can be achieved by using (**C**) caged small molecules, nucleic acids, and proteins or (**D**) engineering naturally light-sensitive proteins.

Light is an ideal stimulus to control biological systems. It acts orthogonally to cellular signals in a large number of organisms and allows for the tightest spatiotemporal control of any input [1]. A major area of research is the use of light to control, arguably, the most fundamental biological pathway, gene expression. This includes methods to control either the transcription (DNA to mRNA) or translation (mRNA to protein) steps (Figure 1). It is possible to either activate or silence each step, or perform both reversibly. Applications of these methods include the control of cell-free systems, gene circuits, and drug/gene delivery. Specific endogenous genes can also be controlled with light by modifying existing technologies.

**Figure 2.**
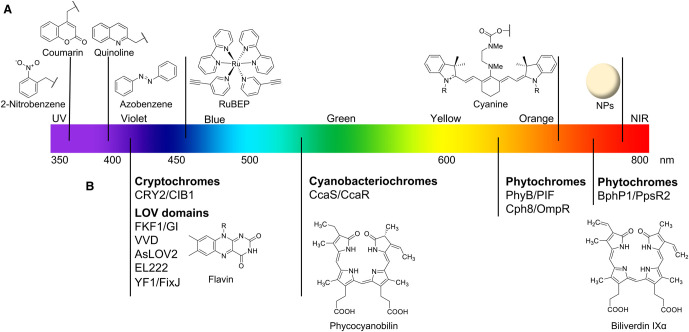
An illustration of the approximate wavelength of activation of a number of photocages (**A**) and engineered naturally light-sensitive proteins (**B**, with chromophores) discussed in this review. NIR = near-infrared, NP = nanoparticle.

Controlling gene expression with light can be approached through both chemical and biological means [2]. One approach is to use chemical photocages, light-sensitive molecules that are linked to a bioactive molecule, blocking its activity. Illumination with a specific wavelength of light causes the photocage to break the linking bond, which reforms the original molecule. Photocages have been attached to small molecule, nucleic acid, or protein regulators of expression or directly to the DNA or mRNA nucleic acid templates themselves [3] (Figure 1A–C). Alternatively, several naturally light-sensitive proteins, which function through multiple different pathways, have been engineered and fused to proteins involved in expression [4] (Figure 1D). Within this review we have covered multiple different approaches that have been used to control gene expression with wavelengths of light from ultraviolet (UV) to near-infrared (NIR) (Figure 2). It is vital to cover multidisciplinary topics to highlight the collective themes and methods used. Simple reporter proteins, such as fluorescent proteins, β-galactosidase, or luciferase, are used as a common way to measure the efficiency of each method. Current applications focus on control of cell-free to *in vivo* gene expression. Human cell lines are widely used to demonstrate application to endogenous pathways. Light-controlled systems are also widely applied *in vivo* to zebrafish, as they are transparent. A few systems have also shown application in mouse models. However, when working *in vivo* it is important proper controls are in place due to the presence of endogenous proteins that respond to light.

## Chemical photocages

The most commonly used photocages are 2-nitrobenzyl derivatives [5] (Figure 2A). They are small, easy to synthesise and are often available commercially; they do, however, cleave in the UV (regularly 365 nm), which can cause cellular damage. Another commonly encountered photocage are coumarin derivatives [5]. These are also irradiated with UV (365–400 nm), but can be chemically modified to allow longer wavelengths of illumination; derivatives absorbing in the green have been prepared [6]. Other photocages that have been used within this review include quinoline (UV irradiation) [7], RuBEP (blue irradiation) [8], and cyanine. Cyanines can be activated up to the NIR [9], but are not often used due to lengthy syntheses. Visible or NIR irradiation is preferable as it causes less cellular damage than UV.

Alternative photocages are azobenzenes and styryl derivatives. They are not photocleavable, but photoswitches, as they change configuration upon irradiation. *Trans*-*Cis* isomerisation of azobenzene and styryl derivatives occurs following UV irradiation [10,11], however modified azobenzenes can be controlled with visible or NIR irradiation [12].

## Light-controlled gene expression using small molecules

Small molecules are widely used to regulate gene expression and have been extensively photocaged to control their activity with light (Figure 2A). The largest group of photocaged molecules are estradiol and tamoxifen analogues, agonists of the estrogen receptor. These have been photocaged with 2-nitrobenzyl-, coumarin- and cyanine-derivatives to control reporter gene expression in mammalian cells [13–16]. Using 2-nitrobenzyl- or thiocoumarin-caged cyclophen, reporter gene expression and phenotype, respectively, could also be controlled in zebrafish [17,18].

A widely used inducer system in mammalian cells is the Tetracycline (Tet) system, under the control of doxycycline. Nitrobenzyl-photocaged doxycyclines have controlled reporter gene expression in mammalian cells [19], tobacco leaves [19] and developing mouse embryos and *xenopus* tadpoles [20]. A number of other small molecule agonists of expression in mammalian cells have been photocaged, including 2-nitrobenzyl-caged nuclear hormones [21] and β-ecdysone [22], as well as a coumarin-caged CREB inhibitor [23].

Carbohydrates are a well-known class of bacterial gene-regulatory molecules. Bacterial gene expression is often placed under control of the lac operator, which is activated using isopropyl-β-d-thiogalactopyranoside (IPTG), a non-hydrolysable allolactose mimic. 2-Nitrobenzyl-photocaged IPTG enabled the efficient regulation of reporter genes and (+)-valencene biosynthesis [24,25]. Arabinose is also widely used to control gene expression via the arabinose operator. 2-Nitrobenzyl-photocaged arabinose was shown to efficiently control expression of Violacein biosynthesis [26].

Nucleotides are vital biological signals and building blocks for transcription. Photocaged analogues are therefore able to control these functions. *In vitro* transcription has been controlled by photocaging adenosine triphosphate (ATP) on the terminal phosphate with a coumarin [27]. This inhibited RNA synthesis until uncaging of ATP with light. In a similar manner, synthetic cells have been activated with 2-nitrobenzyl-caged ATP [28]. Alternatively, uracil and guanine triphosphates, photocaged on the nucleobase with a 2-nitrobenzyl, prevented Watson–Crick base pairing prior to uncaging [29].

Photocaging has also been demonstrated on small molecules that directly interact with DNA or RNA. Toyocamycin is an ATP analogue that controls translation by binding to a ribozyme in mRNA. Light-activated gene expression in mammalian cells was achieved through 2-nitrobenzyl-photocaging [30]. Theophylline is another small molecule used to control translation by binding to an mRNA riboswitch. 2-Nitrobenzyl-photocaged theophylline was used to control expression in bacteria [31]. G-quadruplex (G4) structures are involved in regulation of gene expression. Photocaging the G4-stabilising ligand pyridostatin with a 2-nitrobenzyl allowed the light-activated downregulation of cancer-associated genes in mammalian cells [32]. Cell-free expression can also be controlled via light-activated compaction of DNA/RNA using AzoTAB, which contains an azobenzene photoswitch and has strong nucleic acid affinity in the trans-form and weak in the cis-form [33].

## Light-controlled gene expression using nucleic acids

**Figure 3.**
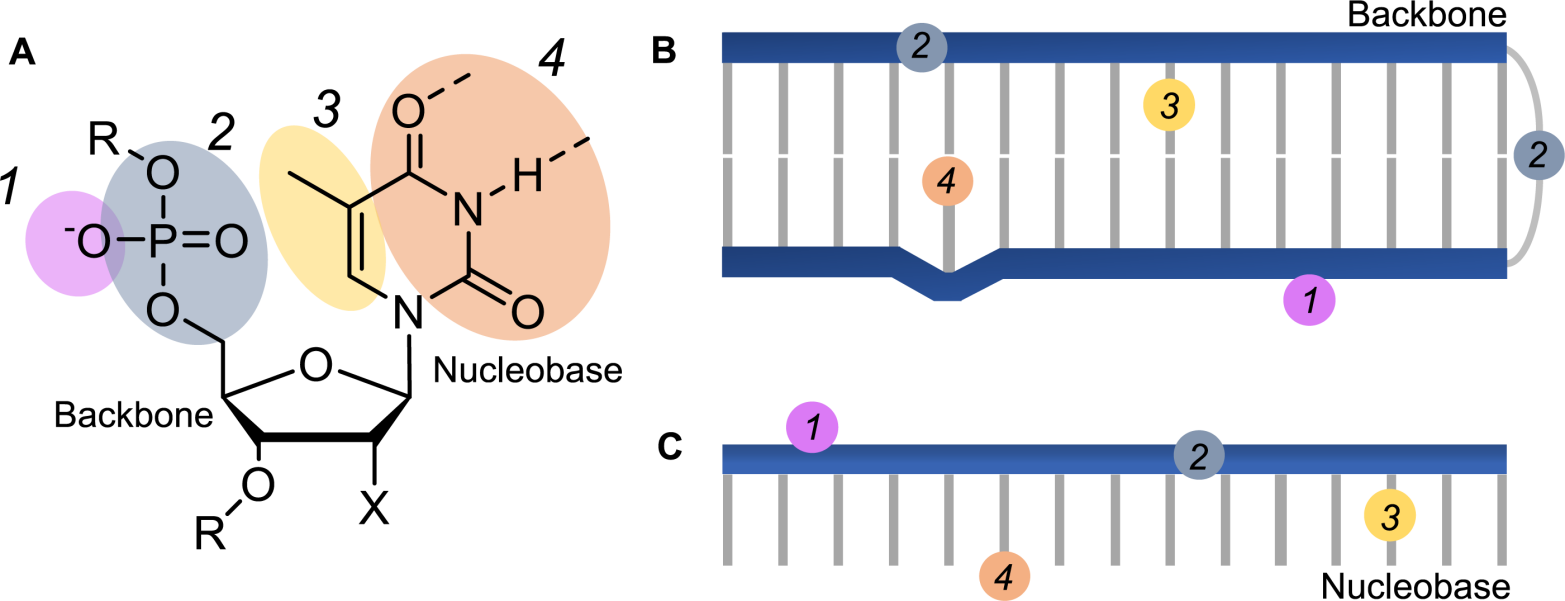
Attachment points of photocages onto DNA (X = H) or RNA (X = OH). (**A**) Photocages have been attached to various positions on DNA/RNA strands to control function, including the phosphate backbone (1), in the backbone (2), on the nucleobase (3), and on the Watson–Crick face of the nucleobase (4). These photocages can be attached to double stranded (**B**) or single stranded (**C**) nucleic acids.

Modification of DNA or RNA with photocages (Figure 2A) can be used to control transcription and translation with light (Figure 3). Generally, light-activated regulation of transcription and translation with nucleic acids can be broadly classified into two categories: the light-induced activation of gene expression (by using the DNA or mRNA template) and the light-induced gene knockdown (via caged antisense oligonucleotides (ASOs) or small interfering RNAs (siRNAs)).

### Gene activation

One approach of inhibiting transcription from a DNA template is by attaching a photocage onto the Watson–Crick face of the nucleobase, impeding base pairing. In this way, incorporation of 2-nitrobenzyl-caged thymidine into a DNA promoter has allowed control of cell-free transcription [34] and reporter gene expression in zebrafish [35]. Alternatively, a covalent inter-strand crosslink was made in a DNA promoter, using a psoralen modified DNA base-pair, to inhibit unwinding of the double helix and therefore transcription [36]. Irradiation initiated cell-free expression through decrosslinking via an adjacent pyrene.

Attachment of photocleavable molecules to other positions on the DNA or RNA can also disrupt recognition of the substrate. One approach is to modify the nucleobase with photocleavable groups at non-Watson–Crick base pairing sites. Amino-modified thymine bases were incorporated into a DNA promoter sequence and reacted with a photocleavable 2-nitrobenzyl biotin to generate a PCR primer, to allow amplification of a gene of interest. Subsequent binding of monovalent streptavidin allowed light-activated cell-free expression. Using this method, expression of protein pores created patterned conductive pathways through synthetic tissues [37,38]. In a similar manner, integration of 2-nitrobenzyl-caged 5-hydroxymethyl(hm)-cytosine and 5-hm-uracil into a DNA template controlled cell-free transcription with light [39]. A photocage was also installed on a nucleobase of a DNA template by using a methyltransferase with a 2-nitrobenzyl-modified S-adenosyl methionine substrate, which was used to control cell-free expression [40]. Enzymatic insertion of photocleavable biotin-coumarin-modified nucleobases into mRNA also enabled controlled expression in mammalian cells [41,42].

Photocages can also be directly attached on the oligonucleotide backbone. A plasmid with a streptavidin/biotin-coumarin backbone-modified promoter was used to control expression in mammalian cells [43]. Coumarin- [44,45] and 2-nitrobenzyl- [46,47] photocages have also been reacted with the phosphate backbone of plasmids and mRNA to allow control of reporter gene expression or phenotype in zebrafish and mammalian cells, and cell-free expression in synthetic cells. Study of head-specific overexpression in zebrafish was achieved using caged mRNA in this way [44].

Photoswitches have also been incorporated into DNA and RNA to reversibly control transcription and translation. By incorporation into a DNA promoter, azobenzenes controlled cell-free transcription [48] and gene expression [49], using UV or blue light as a stimulus. Incorporation of photoswitchable nucleobases into a G4 sequence of a promoter allowed for controlled expression of a reporter protein in zebrafish [50]. Reversible strategies for translation were also developed by capping the 5′ end of the mRNA with azobenzene or styryl photoswitches [50,51]. UV light inhibited the recruitment of a translation initiation factor, while blue light activated recruitment and expression to control differentiation in mammalian cells and zebrafish.

### Gene silencing

ASOs are commonly used to knock down gene expression in cells via targeting complementary mRNA. Base pairing-inhibited 2-nitrobenzyl-caged thymidines have been incorporated into ASOs to photoactivate gene knockdown of developmental genes in zebrafish [52] and cancer-associated genes in mammalian cells [53]. By incorporating a photoreactive nucleobase into an ASO, covalent interstrand crosslinking to its complementary mRNA was achieved in mammalian cells [54].

ASOs can also be light-activated by removal of a complementary inhibitor strand. Inclusion of a 2-nitrobenzyl in the backbone of the complementary strand enabled endogenous gene knockdown and controlled development in zebrafish [55]. The complementary strand has also been attached to the ASO via a 2-nitrobenzyl-containing hairpin, which allowed for photocontrolled knockdown of a cancer-associated gene in mammalian cells [56] and developmental genes in zebrafish [57,58].

ASOs have also been circularised with a photocleavable linker, disrupting mRNA binding until photocleavage. Circularisation, achieved with 2-nitrobenzyl- [59], coumarin- [60], quinoline- [7] and Ru-BEP linkers [8] was applied to control expression with UV or blue light in zebrafish and mammalian cells.

Synthetic siRNAs are double stranded RNA molecules that are widely used to knock down expression of target genes via RNA interference (RNAi). Modification of the phosphate backbone of siRNAs with 2-nitrobenzyl allowed for light-activated knockdown in zebrafish [61]. Similarly, by attaching 2-nitrobenzyl-moieties to the 5′ [62,63] or 5′ and 3′ phosphate termini [64] cell patterning was achieved [65], and the RNAi off-state was improved by adding larger moieties [66]. Attaching an anthracene functionality along with a porphyrin photosensitiser onto the termini of siRNA controlled its function with green or red light [67]. Hybridisation of the siRNA strands was also controlled through incorporation of an azobenzene into the backbone and controlled with red light [68]. 2-Nitrobenzyl-photocaging of the Watson–Crick face of a single nucleobase in siRNA inhibited siRNA:mRNA duplex formation, and thus knocking down a reporter protein [69] and an endogenous gene [70]. An inhibitor of microRNA (miRNA), the natural substrate of RNAi knockdown, has been developed from an RNA hairpin connected through a 2-nitrobenzyl in the backbone [71]. Cleavage with UV allowed binding of the inhibitor to the miRNA, causing altered development in nematodes.

Light-responsive nanoparticles have been extensively used to allow spatiotemporal control of siRNA function. NIR light can release siRNA bound to gold nanoparticles (AuNPs). siRNA attached to AuNPs via terminal thiol-modified siRNA [72–74], DNA bridges [75], or electrostatically [76] enabled NIR-activated knockdown in mammalian cells and therapeutic effects on tumour models *in vivo*. Improved delivery was also accomplished by attaching cell-penetrating peptides to the constructs [73]. NIR-activated knockdown within mammalian cells was also achieved by attachment of siRNA, via a 2-nitrobenzyl, to upconverting nanoparticles (UCNPs), which convert NIR to UV irradiation [77]. Nanoparticles of siRNA have also been formed with positively charged block-copolymers, which aided cellular delivery, containing 2-nitrobenzyls [78].

A number of alterative methods have been used to photocontrol the silencing of gene expression. ASOs have been attached to AuNPs and activated with NIR in mice [79]. Azobenzenes have also been used to reversibly photocontrol binding of an inhibitory hairpin DNA attached to a DNAzyme to activate and deactivate cell-free expression of a reporter gene [80]. Gene knockdown has also been controlled by using a hairpin DNA decoy, modified with photocaged thymidines [81]. Implementing 2-nitrobenzyl-photocaged cytidines in a triplex-forming oligonucleotide also allowed for the light-controlled activation and silencing of gene expression in mammalian cells [82]. Modification of siRNA has also been used to control cell delivery with visible light [83,84].

## Light-controlled gene expression using proteins

Proteins involved in gene expression have been extensively modified, both chemically and with naturally light-sensitive proteins, to control transcription and translation with light. Additionally, by combining these modified proteins with genome targeting technologies, it is possible to light-activate the expression of specific genes on a genome.

### Chemical modification of proteins

Chemically modified proteins can be produced by engineering orthogonal ribosomes and tRNA/tRNA synthetase pairs to accept unnatural amino acids at a TGA codon. This allows precise placement of a photocage within a protein of interest. Using this method, a 2-nitrobenzyl was site-specifically incorporated into an RNA polymerase to demonstrate light-activated gene expression [85] and light-activated RNAi [85] in mammalian cells. Photocages have also been installed into gene editing tools, including Cre recombinases, which enable site specific recombination between DNA sequences called LoxP sites, and Zinc Finger (ZF) nucleases, which are selective towards target DNA sequences. Essential residues in catalytic sites of Cre and ZF nucleases have been modified with 2-nitrobenzyl [86,87] and coumarin [88] to activate expression upon irradiation. Alternatively, incorporation of an azobenzene into the backbone of an epigenetic regulating peptide has allowed photocontrol over its activity in mammalian cells [89].

### Naturally light-sensitive proteins

**Figure 4.**
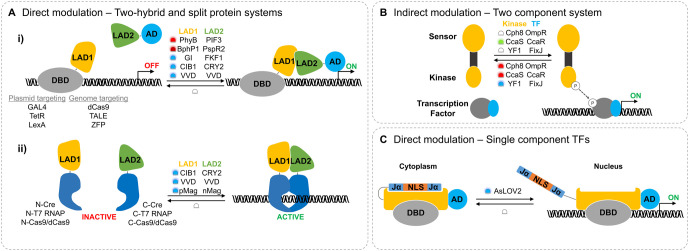
Controlling gene expression by using naturally light-sensitive proteins. (**A**) Protein modules that dimerise in response to light, represented here as Light-Activated Domains (LADs), have been fused to effector domains to create transcription factors that activate gene expression in the presence of light, but are inactivate in the absence of light. Gene expression is activated upon co-localisation of DNA-Binding Domains (DBD) with transActivation Domains (ADs) via a light-activated two-hybrid system (i), or via the co-localisation of inactive C- and N-terminal domains of a split protein and reconstitution of the active protein (ii). (**B**) Light-responsive two component systems (TCSs) are initiated when light is absorbed by the sensory domain of a histidine kinase, which stimulates/represses autophosphorylation of the kinases domains. Phosphorylated kinase domains transfer phosphate groups to downstream Transcription Factors (TF), which can then bind to consensus promoter sequences and activate gene expression. (**C**) Uncaging of the Jα helix in AsLOV2, when exposed to blue light, is used to reveal a shielded Nuclear Localisation Signal (NLS). The exposed NLS is recognised by the importin complex and AsLOV2 is transported into the nucleus. Gene expression can be regulated by fusing DBDs and ADs to AsLOV2, and controlling their nuclear localisation by exposing or shielding the NLS.

In addition to modifying proteins with photocages, naturally light-responsive proteins from plants, cyanobacteria, and algae, have also been incorporated into proteins involved in gene expression (Figure 2B). The mechanisms of these engineered proteins fall into two categories: direct modulation of gene expression using light-activated transcription factors or enzymes, and indirect modulation through light-activated signalling cascades that modulate downstream effectors (Figure 4). While many different photoreceptors have been used to control gene expression, this review focuses primarily on three of the most common classes; phytochromes, Light-Oxygen-Voltage (LOV)-domain proteins, and cryptochromes.

#### Phytochromes (red and NIR light-responsive)

Phytochromes are photoreceptors that utilise tetrapyrrole chromophores such as biliverdin IXα (BV) or phycocyanobilin (PCB) (Figure 2B) to absorb red and NIR light and induce reversible conformational changes in the protein structure. The first photoreceptor used to control gene expression was Phytochrome protein B (PhyB), and its interaction partner Phytochrome-Interacting Factor 3 (PIF3), which dimerise under red light and dissociate under far-red light. By fusing DNA-Binding Domains (DBDs) and transActivation Domains (ADs) to the distinct PhyB and PIF modules, red light inducible two-hybrid systems have been used to activate gene expression in yeast [90] and mammalian cells [91] (Figure 4A-i).

In bacteria, Two-Component signalling Systems (TCSs) comprising of a natural or engineered light-responsive kinase and a downstream response regulator are more prevalent. Cph8 is a light-responsive kinase that was engineered by replacing the osmosensory domain of a membrane-bound histidine kinase, with the Cph1 phytochrome*.* In the absence of light, Cph8 phosphorylates the transcription factor OmpR and promotes gene expression, while in the presence of red light the kinase activity and gene expression are inactivated [92] (Figure 4B). The engineered Cph8/OmpR TCS is analogous to the natural cyanobacteriochrome TCS*,* CcaS/CcaR, which regulates gene expression according to green and red light [93] (Figure 4B). In their most basic forms, these systems were used in bacterial edge detection algorithms [94] and for dual-colour control over gene expression [95]. More efficient versions were later developed by genetic refactoring and mutagenesis [96,97] and have been used to tightly control metabolic flux [98], cell division [99] and feedback loops in bacteria [100].

Most of the red light-responsive gene expression systems require a PCB chromophore to function. Although PCB can be added to the growth media and taken up by cells [90,91], gene cassettes encoding enzymes for PCB biosynthesis are more commonly used to enable cells to synthesise PCB from intracellular heme [92,101,102]. NIR-responsive phytochromes on the other hand utilise BV chromophores, which are produced endogenously by mammalian cells. These NIR-responsive gene expression systems are derived from BphP1 and PpsR2 proteins. PpsR2 is sequestered by BphP1 in NIR light and then released in the presence of red light or absence of light. NIR-responsive transcription factors were created by fusing BphP1 and PpsR2 to DBDs and ADs, and have been shown to activate reporter gene expression in bacteria [103], mammalian cells, and mice [104,105] (Figure 4A-i).

#### LOV domain proteins and Cryptochromes (blue-light responsive)

LOV domain proteins and cryptochromes are two distinct protein families that differ in their protein architecture, yet both use blue light absorbing flavin chromophores (Figure 2B) to induce conformational changes in the protein. Whereas many different LOV domain systems have been developed for blue light-activated gene expression, cryptochrome-based systems are based primarily on the interaction of Cryptochrome protein 2 (CRY2) with Cryptochrome-Interacting Basic-helix-loop-helix protein (CIB1).

The first blue light-responsive transcription system was a TCS implementing an engineered light-sensitive kinase, YF1, created by replacing an oxygen-sensing domain of a natural kinase with the YvtA LOV domain. In the absence of light, YF1 phosphorylated and activated a transcription factor, whereas in the presence of blue-light, kinase activity and gene expression were inactivated [106] (Figure 4B). Based on this system, a bacterial repression switch, pDusk, and activation switch, pDawn [107], were developed. Blue light-regulated gene expression in eukaryotic cells, however, is typically controlled with photoactivatable transcription factors. Flavin-binding, Kelch domain, F-box protein (FKF1) and its interaction partner GIGANTEA (GI) were the first photoreceptor pair to be fused to ADs and DBDs and demonstrated blue light-activated transcription in mammalian cells [108] (Figure 4A-i). Similar light-activated effector proteins based on CRY2/CIB1, and smaller LOV domain proteins also function in this way. Amongst these is Vivid (VVD), a photoreceptor that rapidly and reversibly forms homodimers. CRY2/CIB1 and VVD have been fused to a variety of DBDs and effector domains to control both transcription [109,110] and translation [111,112] in mammalian cells, bacteria [113], and yeast [110,114,115]. They have been applied in various fields including the study of oscillating gene expression [116] and in cell-based immunotherapies [117]. Blue light-induced dimerisation of CRY2/CIB1, VVD, and ‘Magnets’, improved VVD mutants [118], have also been used to control gene expression by reconstituting split enzymes, such as Cre recombinases [110,119,120] and RNA polymerases [121,122] (Figure 4A-ii).

In most cases, light-inducible transcription factors depend on the heterodimerisation of two different proteins to modulate gene expression. However, single component blue light-activated transcription factors also exist, and are typically derived from AsLOV2 or EL222. Upon exposure to blue light, both AsLOV2 and EL222 undergo conformational changes involving the release of an alpha helix from the LOV domain. By inserting nuclear localisation signals (NLS) or nuclear export signals (NES) within the Jα helix of AsLOV2, its cellular location can be switched with blue light (Figure 4C). In this way, genes have been expressed via the import of AsLOV2-based transactivators into the nucleus [123,124], or export of AsLOV2-based repressors into the cytoplasm [125,126]. Alternatively, uncaging of the 4α helix in EL222 is accompanied by the release of a DBD and exposure of a dimerization interface. EL222 fused to an AD has been shown to rapidly induce gene expression in mammalian cells [127] and zebrafish embryos following irradiation [128], and has also been used in yeast to improve their chemical production capabilities [129]. Single component gene expression systems in bacteria [130] and cell-free expression systems [131] have used the transcription factor activity of wild type EL222.

### Genome targeting for light-activated expression

Technologies that target specific sites on the genome allow for precise control of endogenous genes. Genome targeting has been achieved using several types of DNA binding proteins, that can be tailored to recognise specific DNA sequences [132]. These include the Clustered, Regularly Interspaced, Short Palindromic Repeat (CRISPR)-associated nuclease Cas9 system, ZF proteins, Transcription Activator-Like Effectors (TALEs), and recombinases. Photocages and naturally light-sensitive proteins have been incorporated into these technologies for spatiotemporal control of gene knockouts and transcription.

#### CRISPR-Cas9

CRISPR-Cas systems are found in bacteria and archaea as a means of adaptive immunological protection against phages. The bacterial Cas9 nuclease is targeted to the genome using a non-coding guide RNA (gRNA) and cleaves the double stranded DNA causing either a gene knockout or mutagenesis [132]. By mutating Cas9, a catalytically inactive protein (dCas9) can serve as an RNA-guided DNA-binding protein. Both the gRNA and Cas9 have been modified to create light-activated CRISPR-Cas9 systems. A protector DNA, containing 2-nitrobenzyls in the backbone has been bound to gRNA to control gene knockout in mammalian cells [133]. More efficient control was achieved by attaching 2-nitrobenzyls to the Watson–Crick face of nucleobases in the gRNA, which was used to control gene editing in zebrafish embryos [134,135]. To control the Cas9 nuclease, a 2-nitrobenzyl-modified amino acid was installed using an orthogonal tRNA/tRNA synthetase pair [136]. Cas9 has also been covalently linked to UCNPs, via a 2-nitrobenzyl photocage, allowing NIR-activated gene editing and reduction of tumour size in mice [137].

Naturally light-sensitive proteins have been used with both the Cas9 nuclease and dCas9 to achieve light-activated gene knockout and transcriptional control, respectively (Figure 4A). These rely on the dimerization of split Cas9 domains and/or dCas9 with ADs via photoreceptors, particularly CRY2/CIB1 [138–140] and magnet proteins [140,141]. Other light-activated CRISPR-Cas9 systems depend on light-activated phosphorylation [142] or cyclic diguanylate monophosphate (c-di-GMP) signalling cascades [143], as well as a dimeric green fluorescent protein, pdDronpa [144].

#### Zinc finger and transcription activator-like effector proteins

ZF proteins recognise a specific 3-base pair DNA sequence and individual TALE proteins each recognise a single base pair. Hence, effector proteins can be targeted to specific locations on a genome by fusing them to contiguous ZF and TALE domains [132]. By fusing ZFs to GI and an AD to FKF1, gene expression in mammalian cells has been controlled [145] (Figure 4A-i). TALEs and ADs using the CRY2/CIB1 pair have been used to regulate gene expression in mouse brain cortex [146] and recruitment of epigenetic modifiers in rat neuronal stem cells [147] (Figure 4A-i).

#### Recombinases

In recombinase-mediated gene editing, DNA flanked by LoxP recognition sites is commonly excised from the genome of transgenic organisms to study the response of gene knockouts [148]. By incorporating these artificial recognition sites in the genome, light-activated recombinases have been used to control the expression of targeted genes. Light-activated Cre systems have been developed using 2-nitrobenzyl-photocaged tamoxifen [149], which controlled light-dependent recombination and gene expression in mice [150]. CRY2/CIB1, magnet, or VVD pairs have also been fused to split Cre and Flp recombinase domains to control gene expression in mouse brains [151–154] and zebrafish embryos [153] (Figure 4A-ii). This has been applied to reconstruct the morphology of single neurons across an entire mouse brain [153].

## Outlook

Controlling gene expression with light has led to a wide range of applications in cell-free to *in vivo* systems. Chemical modification of nucleic acids with photocages has been used to create conductive pathways in synthetic tissues [37,38] and control the development of zebrafish [7,55,60,134,135] and cancer therapeutics [75,76,79,137] with light. While naturally light-sensitive proteins have been used to control cell division [99], bioreactors [129], and genome activation in zebrafish and mice [146,151,153]. Identification of new applications is vital to realising the full potential of light-controlled gene expression.

As we have focused on discussing methods and applications within this review, there has been a limited discussion of the efficiencies of each system. To allow general use of these technologies an efficient ‘ON’ and ‘OFF’ state is required, where minimal activity is observed without light, and maximal with light. It is worth noting the efficiencies vary wildly for the methods discussed. More efficient and easily accessible systems are required to allow more general use.

Photocages and naturally light-sensitive proteins each have their own advantages and limitations. Plasmid DNA encoding light-sensitive proteins and some simple photocages attached to DNA are commercially available. Most photocaged small molecules and the more advanced photocages must be synthesised in house. However, this is also an advantage as more diverse systems have been generated through the chemical synthesis of photocages with small molecules, nucleic acids, and proteins. Another advantage of photocages is their immediate generation of an active species, compared with the requirement that cells first generate the light-sensitive proteins from plasmid DNA, prior to their application. Most chemical photocages absorb in the UV, which can damage cells. Chemical photocages that absorb at longer wavelengths of light, which minimises cellular damage and increases tissue penetration [155], are becoming more popular. However, more easily accessible NIR photocages would give a major boost to this area. In contrast with this, naturally light-sensitive proteins regularly absorb UV, visible or NIR light. Hence, more orthogonal systems also exist for light-sensitive proteins, where expression of different genes can be put under the control of different wavelengths of light, even inside the same cell. Additionally, many reversible naturally light-sensitive proteins exist, compared with only a few chemical photoswitches. Regarding cell delivery, small molecules are highly advantageous as they are cell permeable. Plasmid DNA encoding naturally light-sensitive proteins and photocaged nucleic acids must be transfected. Naturally light-sensitive proteins also tend to require multiple or complex plasmids to be delivered. However, in bacteria and yeast these systems can be controlled over multiple generations as the plasmid system encoding the light-sensitive proteins is replicated. An important goal for this field is to combine the light-activation systems with cell delivery systems, where only limited examples exist [53,72,73,78,146,151,153], to produce far-reaching technologies.

## Perspective

- **Importance:** Using light to control gene expression opens the door to myriad biotechnology applications in therapeutics, biomanufacturing, and emerging fields such as cell-free systems.
- **Summary:** Control of exogenous and endogenous gene expression has been achieved in many different ways. Chemical tools rely on attaching photocages to small molecules, nucleic acids, and proteins, while biological tools use engineered naturally light-sensitive proteins.
- **Direction:** Beyond identifying new applications, the three main areas of research that still require attention are: increased usage of longer wavelength photocages, more efficient and general systems, and combining light-activation with cell delivery.

## Open Access

Open access for this article was enabled by the participation of University of Oxford in an all-inclusive *Read & Publish* pilot with Portland Press and the Biochemical Society under a transformative agreement with JISC.

## Abbreviations

AD: transactivation domain
ASO: antisense oligonucleotide
ATP: adenosine triphosphate
AuNP: gold nanoparticle
BV: biliverdin Ixα
c-di-GMP: cyclic diguanylate monophosphate
CIB1: cryptochrome-interacting basic helix-loop-helix protein
CREB: cAMP response element-binding protein
CRISPR: clustered, regularly interspaced, short palindromic repeat
CRY2: cryptochrome protein 2
DBD: DNA binding domain
dCas9: deactivated Cas9
DNA: deoxyribonucleic acid
FKF1: Flavin-binding, Kelch domain, F-box protein
G4: G-quadruplex
GI: GIGANTEA
gRNA: guide RNA
hm: hydroxymethyl
IPTG: isopropyl-β-D-thiogalactopyranoside
LAD: light-activated domain
LOV: light-oxygen-voltage
miRNA: microRNA
mRNA: messenger RNA
NIR: near-infrared
NLS: nuclear localisation signal
NP: nanoparticle
PCB: phycocyanobilin
PCR: polymerase chain reaction
PhyB: phytochrome protein B
PIF3: phytochrome interacting factor 3
RNA: ribonucleic acid
RNAi: RNA interference
siRNA: small interfering RNA
TALE: transcription activator-like effectors
TCS: two component system
Tet: tetracycline
TF: transcription factor
tRNA: transfer RNA
UCNP: upconverting nanoparticle
UV: ultraviolet
VVD: Vivid
ZF: zinc finger
